# Supplementary material for: Modified Albumin–Bilirubin Model for Stratifying Survival in Patients with Hepatocellular Carcinoma Receiving Anticancer Therapy
Source: Cancers (Basel). 2022 Oct 17;14(20):5083. doi: 10.3390/cancers14205083 (PMC9600636; doi:10.3390/cancers14205083)
Supplement: Supplementary file 1 [file cancers-14-05083-s001.zip › cancers-1948419-supplementary.pdf]

Supplementary Materials

Modified albumin–bilirubin model for stratifying survival in patients with hepatocellular carcinoma receiving anticancer therapy

Wei-Fan Hsu, Hung-Wei Wang, Shih-Chao Hsu, Te-Hong Chen, Chien-Hung Lin, Ying-Chun Lin, Yu-Wei Chang, Yu-Min Liao, Hsueh-Chou Lai, Cheng-Yuan Peng

Table S1. Demographics, baseline characteristics, and outcomes of enrolled and excluded patients

n (%) or median (IQR)	Eligible for investigation (*n* = 2743)	Enrolled (*n* = 2116)	Excluded (*n* = 627)	*p* Value
Age (years)	64 (56–72)	64 (56–72)	62 (55–73)	0.258
Sex (male), *n* (%)	1973 (71.9)	1513 (71.5)	460 (73.4)	0.362
Follow-up months	18.09 (4.80–45.29)	23.23 (8.22–51.08)	3.17 (1.22–16.28)	< 0.001
Platelet count (×10^9/L)	156 (109–213)	157 (110–214)	142 (94–209)	0.049
AST (U/L)	50 (32–84)	49 (32–84)	62 (40–87)	0.001
ALT (U/L)	41 (27–65)	41 (26–64)	46 (29–70)	0.043
Total bilirubin (mg/dL)	1.0 (0.7–1.5)	0.9 (0.6–1.2)	1.7 (1.0–3.6)	< 0.001
Albumin (g/dL)	3.9 (3.3–4.3)	4.0 (3.6–4.4)	3.2 (2.8–3.9)	< 0.001
INR	1.09 (1.03–1.18)	1.08 (1.02–1.14)	1.19 (1.11–1..6)	< 0.001
Creatinine (mg/dL)	0.91 (0.76–1.11)	0.91 (0.76–1.11)	0.89 (0.74–1.13)	0.474
Etiology				
Alcohol, *n* (%)	553 (20.2)	428 (20.2)	125 (19.9)	0.227
HBV, *n* (%)	1375 (50.1)	1073 (50.7)	302 (48.2)	0.339
HCV, *n* (%)	1051 (38.3)	847 (40.0)	204 (32.5)	0.002
Diabetes mellitus, *n* (%)	907 (33.1)	781 (36.9)	126 (20.1)	0.338
Liver cirrhosis, *n* (%)	1712 (62.4)	1227 (58.0)	485 (77.4)	< 0.001
Child–Pugh score	5 (5–7)	5 (5–6)	8 (5–10)	< 0.001
Class A/B/C, *n* (%)	2026/540/175 (73.9/19.7/6.4)	1808/308/0 (85.4/14.6/0)	218/232/175 (34.8/37.0/27.9)	< 0.001

FIB-4	3.27 (1.98–5.80)	3.22 (1.97–5.72)	4.26 (2.20–6.43)	0.005
ALBI score	-2.52 (-2.94– -1.94)	-2.66 (-3.02– -2.20)	-1.73 (-2.40– -1.23)	< 0.001
BCLC stage A/B/C/D, *n* (%)	1071/709/721/239 (39.1/25.9/26.3/8.7)	968/590/558/0 (45.7/27.9/26.4/0)	103/119/163/239 (16.5/19.1/26.1/38.3)	< 0.001
AFP (ng/mL)	34.71 (5.99–817.54)	30.24 (5.70–491.17)	68.89 (7.59–2958.70)	< 0.001
AFP ≥ 400 ng/mL, *n* (%)	805 (29.3)	564 (26.7)	241 (38.4)	< 0.001
Max. tumor size (cm)	4.2 (2.3–8.0)	3.8 (2.2–7.5)	6.0 (3.0–10.0)	< 0.001
Tumor volume > 50%, *n* (%)	501 (18.3)	302 (14.3)	199 (31.7)	< 0.001
MVI, *n* (%)	674 (24.6)	427 (20.2)	247 (39.4)	< 0.001
MELD score	8 (7–11)	8 (7–10)	9 (7–12)	0.001
CLIP score	1 (0–3)	1 (0–2)	3 (1–4)	< 0.001
Therapy				< 0.001
Surgical resection, *n* (%)	630 (23.0)	629 (29.7)	1 (0.2)	
RFA, *n* (%)	419 (15.3)	413 (19.5)	6 (1.0)	
TACE, *n* (%)	817 (29.8)	799 (37.8)	18 (2.9)	
Systemic therapy, *n* (%)	174 (6.3)	159 (7.5)	15 (2.4)	
Radiotherapy, *n* (%)	74 (2.7)	70 (3.3)	4 (0.6)	
TACE + radiotherapy, *n* (%)	47 (1.7)	46 (2.2)	1 (0.2)	
Liver transplantation, *n* (%)	143 (5.2)	0 (0)	143 (22.8)	
Hospice or transfer, *n* (%)	439 (16.0)	0 (0)	439 (70.0)	

Overall survival (months)[a]	33.41 (29.61–37.21)	44.94 (39.54–50.34)	6.11 (4.88–7.34)	< 0.001

Data are presented as medians (first quartile to third quartile)

[a] Data are presented as medians (95% confidence interval)

Abbreviations: AFP, α-fetoprotein; ALBI, albumin–bilirubin; ALT, alanine aminotransferase; AST, aspartate aminotransferase; BCLC, Barcelona Clinic Liver Cancer; CLIP, Cancer of the Liver Italian Program; FIB-4, fibrosis-4; HBV, hepatitis B virus; HCV, hepatitis C virus; INR, international normalized ratio; IQR, interquartile range; MELD, model for end-stage liver disease; MVI, macrovascular invasion; RFA, radiofrequency ablation; TACE, transarterial chemoembolization

Table S2. Univariate and multivariate Cox regression analyses of factors associated with overall survival in patients with BCLC stage A

Variable	Univariate analysis		Multivariable analysis 1		Multivariable analysis 2	
	HR (95% CI)	*p* Value	HR (95% CI)	*p* Value	HR (95% CI)	*p* Value
Age (years)	1.026 (1.014–1.038)	< 0.001	1.020 (1.008–1.033)	0.001	1.023 (1.010–1.036)	< 0.001
Sex: male vs. female	0.739 (0.619–1.016)	0.066				
DM: yes vs. no	1.469 (1.154–1.870)	0.002	1.382 (1.083–1.763)	0.009	1.295 (1.014–1.654)	0.038
Platelet count ($\times 10^9$/L) ≥ 100 vs. < 100	0.385 (0.301–0.491)	< 0.001	0.586 (0.447–0.767)	< 0.001	0.528 (0.403–0.691)	< 0.001
ALT (U/L) > 40 vs. ≤ 40	1.285 (1.008–1.637)	0.043				
AFP (ng/mL) ≥ 400 vs. < 400	1.968 (1.419–2.729)	< 0.001	2.382 (1.713–3.313)	< 0.001	2.295 (1.650–3.191)	< 0.001
Tumor size (cm)	1.062 (0.955–1.181)	0.264				
ALBI grade 2 vs. 1	3.203 (2.496–4.110)	< 0.001	2.486 (1.888–3.272)	< 0.001		
ALBI grade 3 vs. 1	5.114 (2.815–9.292)	< 0.001	3.955 (2.128–7.351)	< 0.001		
ALBI grade 3 vs. 2	1.597 (0.885–2.881)	0.120	1.591 (0.876–2.889)	0.127		
mALBI grade 2 vs. 1	2.167 (1.551–3.027)	< 0.001			1.564 (1.099–2.225)	0.013
mALBI grade 3 vs. 1	5.679 (3.854–8.369)	< 0.001			3.403 (2.219–5.219)	< 0.001
mALBI grade 3 vs. 2	2.621 (1.952–3.519)	< 0.001			2.176 (1.590–2.978)	< 0.001

Abbreviations: AFP, α-fetoprotein; ALBI, albumin–bilirubin; ALT, alanine aminotransferase; BCLC, Barcelona Clinic Liver Cancer; CI, confidence interval; DM, diabetes mellitus; HR, hazard ratio; mALBI, modified albumin–bilirubin

Table S3. Univariate and multivariate Cox regression analyses of factors associated with overall survival in patients with BCLC stage B

Variable	Univariate analysis		Multivariable analysis 1		Multivariable analysis 2	
	HR (95% CI)	*p* Value	HR (95% CI)	*p* Value	HR (95% CI)	*p* Value
Age (years)	1.016 (1.006–1.025)	0.001	1.016 (1.007–1.025)	0.001	1.016 (1.006–1.025)	0.001
Sex: male vs. female	0.986 (0.767–1.268)	0.914				
DM: yes vs. no	1.060 (0.839–1.339)	0.626				
Platelet count ($\times 10^9$/L) ≥ 100 vs. < 100	0.863 (0.657–1.133)	0.288				
ALT (U/L) > 40 vs. ≤ 40	1.162 (0.929–1.454)	0.189				
AFP (ng/mL) ≥ 400 vs. < 400	1.875 (1.480–2.376)	< 0.001	1.784 (1.398–2.276)	< 0.001	1.766 (1.387–2.247)	< 0.001
Tumor size (cm)	1.079 (1.051–1.108)	< 0.001	1.077 (1.048–1.107)	< 0.001	1.079 (1.050–1.108)	< 0.001
ALBI grade 2 vs. 1	2.260 (1.788–2.858)	< 0.001	2.102 (1.656–2.668)	< 0.001		
ALBI grade 3 vs. 1	3.684 (2.338–5.805)	< 0.001	3.771 (2.386–5.960)	< 0.001		
ALBI grade 3 vs. 2	1.630 (1.044–2.545)	0.032	1.795 (1.145–2.812)	0.011		
mALBI grade 2 vs. 1	1.981 (1.455–2.697)	< 0.001			1.801 (1.312–2.471)	< 0.001
mALBI grade 3 vs. 1	3.807 (2.700–5.368)	< 0.001			3.567 (2.520–5.050)	< 0.001
mALBI grade 3 vs. 2	1.921 (1.483–2.490)	< 0.001			1.981 (1.522–2.579)	< 0.001

Abbreviations: AFP, α-fetoprotein; ALBI, albumin–bilirubin; ALT, alanine aminotransferase; BCLC, Barcelona Clinic Liver Cancer; CI, confidence interval; DM, diabetes mellitus; HR, hazard ratio; mALBI, modified albumin–bilirubin

Table S4. Univariate and multivariate Cox regression analyses of factors associated with overall survival in patients with BCLC stage C

Variable	Univariate analysis		Multivariable analysis 1		Multivariable analysis 2	
	HR (95% CI)	*p* Value	HR (95% CI)	*p* Value	HR (95% CI)	*p* Value
Age (years)	0.992 (0.984–1.000)	0.043				
Sex: male vs. female	1.113 (0.882–1.406)	0.367				
DM: yes vs. no	0.909 (0.747–1.107)	0.342				
Platelet count ($\times 10^9$/L) ≥ 100 vs. < 100	1.190 (0.884–1.603)	0.252				
ALT (U/L) > 40 vs. ≤ 40	1.069 (0.883–1.295)	0.492				
AFP (ng/mL) ≥ 400 vs. < 400	1.690 (1.399–2.042)	< 0.001	1.670 (1.376–2.027)	< 0.001	1.603 (1.322–1.943)	< 0.001
Tumor size (cm)	1.010 (1.002–1.017)	0.011				
MVI: yes vs. no	1.568 (1.258–1.953)	< 0.001	1.462 (1.169–1.827)	0.001	1.500 (1.200–1.874)	< 0.001
ALBI grade 2 vs. 1	1.680 (1.376–2.052)	< 0.001	1.735 (1.413–2.131)	< 0.001		
ALBI grade 3 vs. 1	2.698 (1.651–4.412)	< 0.001	2.887 (1.735–4.803)	< 0.001		
ALBI grade 3 vs. 2	1.606 (0.996–2.588)	0.052	1.664 (1.015–2.727)	0.044		
mALBI grade 2 vs. 1	1.416 (1.053–1.903)	.021			1.471 (1.092–1.982)	0.011
mALBI grade 3 vs. 1	2.399 (1.758–3.274)	< .001			2.543 (1.853–3.490)	< 0.001
mALBI grade 3 vs. 2	1.695 (1.384–2.076)	< .001			1.728 (1.407–2.124)	< 0.001

Abbreviations: AFP, α-fetoprotein; ALBI, albumin–bilirubin; ALT, alanine aminotransferase; BCLC, Barcelona Clinic Liver Cancer; CI, confidence interval; DM, diabetes mellitus; HR, hazard ratio; mALBI, modified albumin–bilirubin; MVI, macrovascular invasion

Table S5. Univariate and multivariate Cox regression analyses of factors associated with overall survival in patients receiving surgical resection

Variable	Univariate analysis		Multivariable analysis 1		Multivariable analysis 2	
	HR (95% CI)	*p* Value	HR (95% CI)	*p* Value	HR (95% CI)	*p* Value
Age (years)	1.005 (0.991–1.020)	0.480				
Sex: male vs. female	1.331 (0.891–1.988)	0.163				
DM: yes vs. no	1.079 (0.770–1.511)	0.660				
Platelet count ($\times 10^9$/L) ≥ 100 vs. < 100	0.644 (0.364–1.139)	0.130				
ALT (U/L) > 40 vs. ≤ 40	1.061 (0.766–1.470)	0.720				
AFP (ng/mL) ≥ 400 vs. < 400	2.932 (2.077–4.138)	< 0.001	2.016 (1.386–2.933)	< 0.001	2.188 (1.513–3.164)	< 0.001
Tumor size (cm)	1.142 (1.108–1.176)	< 0.001	1.101 (1.060–1.144)	< 0.001	1.103 (1.064–1.144)	< 0.001
MVI: yes vs. no	3.016 (1.818–5.005)	< 0.001	1.938 (1.145–3.281)	0.014	1.800 (1.066–3.041)	0.028
ALBI grade 2 vs. 1	2.427 (1.679–3.510)	< 0.001	1.713 (1.152–2.545)	0.008		
ALBI grade 3 vs. 1	4.384 (1.390–13.830)	0.012	4.827 (1.523–15.295)	0.007		
ALBI grade 3 vs. 2	1.806 (0.558–5.850)	0.324	2.819 (0.862–9.214)	0.086		
mALBI grade 2 vs. 1	1.686 (1.189–2.390)	0.003			1.452 (1.017–2.074)	0.040
mALBI grade 3 vs. 1	3.458 (1.843–6.486)	< 0.001			2.860 (1.501–5.451)	0.001
mALBI grade 3 vs. 2	2.051 (1.119–3.757)	0.020			1.970 (1.068–3.632)	0.030

Abbreviations: AFP, α-fetoprotein; ALBI, albumin–bilirubin; ALT, alanine aminotransferase; CI, confidence interval; DM, diabetes mellitus; HR, hazard ratio; mALBI, modified albumin–bilirubin; MVI, macrovascular invasion

Table S6. Univariate and multivariate Cox regression analyses of factors associated with overall survival in patients receiving RFA

Variable	Univariate analysis		Multivariable analysis 1		Multivariable analysis 2	
	HR (95% CI)	*p* Value	HR (95% CI)	*p* Value	HR (95% CI)	*p* Value
Age (years)	1.006 (0.990–1.023)	0.462				
Sex: male vs. female	1.073 (0.747–1.540)	0.704				
DM: yes vs. no	1.245 (0.886–1.749)	0.207				
Platelet count ($\times 10^9$/L) ≥ 100 vs. < 100	0.479 (0.340–0.673)	< 0.001	0.678 (0.466–0.986)	0.042	0.640 (0.440–0.932)	0.020
ALT (U/L) > 40 vs. ≤ 40	1.205 (0.856–1.699)	0.285				
AFP (ng/mL) ≥ 400 vs. < 400	2.904 (1.763–4.785)	< 0.001	2.484 (1.449–4.256)	0.001	2.295 (1.321–3.988)	0.003
Tumor size (cm)	1.442 (1.276–1.629)	< 0.001	1.381 (1.215–1.570)	< 0.001	1.394 (1.230–1.579)	< 0.001
MVI: yes vs. no	1.886 (0.466–7.635)	0.374				
ALBI grade 2 vs. 1	2.323 (1.621–3.328)	< 0.001	2.037 (1.379–3.010)	< 0.001		
ALBI grade 3 vs. 1	4.181 (1.977–8.844)	< 0.001	3.437 (1.518–7.784)	0.003		
ALBI grade 3 vs. 2	1.800 (0.868–3.733)	0.114	1.687 (0.775–3.671)	0.187		
mALBI grade 2 vs. 1	1.699 (1.008–2.863)	0.047			1.556 (0.896–2.703)	0.117
mALBI grade 3 vs. 1	3.563 (1.983–6.403)	< 0.001			2.974 (1.536–5.760)	0.001
mALBI grade 3 vs. 2	2.097 (1.400–3.142)	< 0.001			1.911 (1.235–2.959)	0.004

Abbreviations: AFP, α-fetoprotein; ALBI, albumin–bilirubin; ALT, alanine aminotransferase; CI, confidence interval; DM, diabetes mellitus; HR, hazard ratio; mALBI, modified albumin–bilirubin; MVI, macrovascular invasion; RFA, radiofrequency ablation

Table S7. Univariate and multivariate Cox regression analyses of factors associated with overall survival in patients receiving TACE

Variable	Univariate analysis		Multivariable analysis 1		Multivariable analysis 2	
	HR (95% CI)	*p* Value	HR (95% CI)	*p* Value	HR (95% CI)	*p* Value
Age (years)	0.991 (0.984–0.998)	0.009				
Sex: male vs. female	1.171 (0.970–1.413)	0.101				
DM: yes vs. no	1.016 (0.850–1.214)	0.864				
Platelet count ($\times 10^9$/L) ≥ 100 vs. < 100	1.325 (1.082–1.622)	0.006	1.321 (1.065–1.637)	0.011	1.320 (1.067–1.634)	0.011
ALT (U/L) > 40 vs. ≤ 40	1.166 (0.979–1.389)	0.085				
AFP (ng/mL) ≥ 400 vs. < 400	2.138 (1.791–2.551)	< 0.001	1.949 (1.620–2.346)	< 0.001	1.828 (1.520–2.198)	< 0.001
Tumor size (cm)	1.020 (1.014–1.026)	< 0.001	1.008 (1.001–1.016)	0.032	1.011 (1.003–1.019)	0.005
MVI: yes vs. no	2.753 (2.270–3.339)	< 0.001	2.189 (1.773–2.702)	< 0.001	2.232 (1.809–2.753)	< 0.001
ALBI grade 2 vs. 1	1.664 (1.378–2.011)	< 0.001	1.748 (1.429–2.137)	< 0.001		
ALBI grade 3 vs. 1	2.462 (1.684–3.601)	< 0.001	3.142 (2.137–4.621)	< 0.001		
ALBI grade 3 vs. 2	1.480 (1.028–2.128)	0.035	1.798 (1.241–2.606)	0.002		
mALBI grade 2 vs. 1	1.404 (1.068–1.847)	0.015			1.568 (1.178–2.088)	0.002
mALBI grade 3 vs. 1	2.478 (1.857–3.306)	< 0.001			2.830 (2.095–3.822)	< 0.001
mALBI grade 3 vs. 2	1.764 (1.460–2.133)	< 0.001			1.804 (1.483–2.195)	< 0.001

Abbreviations: AFP, α-fetoprotein; ALBI, albumin–bilirubin; ALT, alanine aminotransferase; CI, confidence interval; DM, diabetes mellitus; HR, hazard ratio; mALBI, modified albumin–bilirubin; MVI, macrovascular invasion; TACE, transarterial chemoembolization

Table S8. Univariate and multivariate Cox regression analyses of factors associated with overall survival in patients receiving systemic therapy

Variable	Univariate analysis		Multivariable analysis 1		Multivariable analysis 2	
	HR (95% CI)	*p* Value	HR (95% CI)	*p* Value	HR (95% CI)	*p* Value
Age (years)	0.996 (0.980–1.012)	0.623				
Sex: male vs. female	1.464 (0.881–2.431)	0.141				
DM: yes vs. no	0.940 (0.651–1.356)	0.739				
Platelet count ($\times 10^9$/L) ≥ 100 vs. < 100	1.611 (0.923–2.811)	0.094				
ALT (U/L) > 40 vs. ≤ 40	1.351 (0.928–1.967)	0.116				
AFP (ng/mL) ≥ 400 vs. < 400	1.327 (0.939–1.876)	0.109				
Tumor size (cm)	1.072 (1.033–1.113)	< 0.001	1.052 (1.010–1.095)	0.015	1.051 (1.009–1.095)	0.016
MVI: yes vs. no	1.627 (1.099–2.409)	0.015				
ALBI grade 2 vs. 1	2.013 (1.335–3.034)	0.001	1.577 (0.986–2.522)	0.057		
ALBI grade 3 vs. 1	6.923 (2.544–18.841)	< 0.001	5.926 (1.964–17.880)	0.002		
ALBI grade 3 vs. 2	3.440 (1.340–8.830)	0.010	3.757 (1.309–10.782)	0.014		
mALBI grade 2 vs. 1	1.403 (0.698–2.817)	0.342			1.491 (0.691–3.218)	0.309
mALBI grade 3 vs. 1	2.543 (1.243–5.199)	0.011			2.283 (1.055–4.939)	0.036
mALBI grade 3 vs. 2	1.813 (1.255–2.618)	0.002			1.531 (1.035–2.265)	0.033

Abbreviations: AFP, α-fetoprotein; ALBI, albumin–bilirubin; ALT, alanine aminotransferase; CI, confidence interval; DM, diabetes mellitus; HR, hazard ratio; mALBI, modified albumin–bilirubin; MVI, macrovascular invasion

Table S9. Univariate and multivariate Cox regression analyses of factors associated with overall survival in patients with Child–Pugh class A

Variable	Univariate analysis		Multivariable analysis 1		Multivariable analysis 2	
	HR (95% CI)	*p* Value	HR (95% CI)	*p* Value	HR (95% CI)	*p* Value
Age (years)	0.996 (0.990–1.002)	0.204				
Sex: male vs. female	1.168 (0.997–1.367)	0.054				
DM: yes vs. no	0.981 (0.849–1.134)	0.799				
Platelet count ($\times 10^9$/L) ≥ 100 vs. < 100	0.946 (0.792–1.131)	0.544				
ALT (U/L) > 40 vs. ≤ 40	1.394 (1.210–1.607)	< 0.001				
AFP (ng/mL) ≥ 400 vs. < 400	3.050 (2.637–3.527)	< 0.001	2.286 (1.960–2.668)	< 0.001	2.219 (1.900–2.592)	< 0.001
Tumor size (cm)	1.032 (1.028–1.037)	< 0.001	1.013 (1.007–1.020)	< 0.001	1.017 (1.010–1.023)	< 0.001
MVI: yes vs. no	4.784 (4.090–5.595)	< 0.001	3.032 (2.540–3.619)	< 0.001	3.143 (2.630–3.755)	< 0.001
ALBI grade 2 vs. 1	2.435 (2.115–2.804)	< 0.001	2.110 (1.826–2.439)	< 0.001		
ALBI grade 3 vs. 1	5.872 (2.429–14.195)	< 0.001	5.789 (2.389–14.029)	< 0.001		
ALBI grade 3 vs. 2	2.411 (0.998–5.826)	0.051	2.744 (1.132–6.651)	0.025		
mALBI grade 2 vs. 1	2.005 (1.673–2.404)	< 0.001			1.825 (1.518–2.194)	< 0.001
mALBI grade 3 vs. 1	4.071 (3.158–5.246)	< 0.001			3.524 (2.724–4.557)	< 0.001
mALBI grade 3 vs. 2	2.030 (1.639–2.514)	< 0.001			1.931 (1.556–2.397)	< 0.001

Abbreviations: AFP, α-fetoprotein; ALBI, albumin–bilirubin; ALT, alanine aminotransferase; CI, confidence interval; DM, diabetes mellitus; HR, hazard ratio; mALBI, modified albumin–bilirubin; MVI, macrovascular invasion

Table S10. Correlation between Child–Pugh score or class and modified ALBI grade or original ALBI grade

	Child–Pugh score	Child–Pugh class
ALBI grade	0.609	0.681
mALBI grade	0.636	0.827

Abbreviations: ALBI, albumin–bilirubin; mALBI, modified albumin–bilirubin

Table S11. Estimated 95% confidence intervals for the coefficients of ALBI and mALBI grades determined through bootstrapping in multivariate Cox regression analyses for different subgroups

Subgroup	Variable	B (95% CI)	*p* Value
All enrolled patients	ALBI grade 2 vs. 1	0.863 (0.705–1.022)	< 0.001
	ALBI grade 3 vs. 1	1.434 (1.104–1.783)	< 0.001
	ALBI grade 3 vs. 2	0.571 (0.287–0.877)	< 0.001
	mALBI grade 2 vs. 1	0.632 (0.438–0.891)	< 0.001
	mALBI grade 3 vs. 1	1.386 (1.163–1.654)	< 0.001
	mALBI grade 3 vs. 2	0.754 (0.577–0.917)	< 0.001
Curative therapy	ALBI grade 2 vs. 1	0.671 (0.382–0.970)	< 0.001
	ALBI grade 3 vs. 1	1.243 (0.394–2.020)	0.004
	ALBI grade 3 vs. 2	0.573 (-0.206–1.329)	0.120
	mALBI grade 2 vs. 1	0.426 (0.136–0.724)	0.004
	mALBI grade 3 vs. 1	1.017 (0.596–1.461)	< 0.001
	mALBI grade 3 vs. 2	0.591 (0.212–0.914)	0.001
TACE	ALBI grade 2 vs. 1	0.558 (0.336–0.791)	< 0.001
	ALBI grade 3 vs. 1	1.145 (0.733–1.552)	< 0.001
	ALBI grade 3 vs. 2	0.587 (0.226–0.972)	0.002
	mALBI grade 2 vs. 1	0.450 (0.136–0.873)	0.001
	mALBI grade 3 vs. 1	1.040 (0.710–1.475)	< 0.001
	mALBI grade 3 vs. 2	0.590 (0.394–0.804)	< 0.001
Systemic therapy	ALBI grade 2 vs. 1	0.456 (0.038–1.113)	0.039
	ALBI grade 3 vs. 1	1.779 (0.342–2.981)	0.002
	ALBI grade 3 vs. 2	1.324 (-0.056–2.470)	0.009
	mALBI grade 2 vs. 1	0.399 (-0.260–1.165)	0.194
	mALBI grade 3 vs. 1	0.825 (0.139–1.607)	0.009
	mALBI grade 3 vs. 2	0.426 (0.052–0.815)	0.032
Child–Pugh class A	ALBI grade 2 vs. 1	0.747 (0.558–0.907)	0.001
	ALBI grade 3 vs. 1	1.756 (0.890–4.589)	0.036
	ALBI grade 3 vs. 2	1.009 (0.034–4.393)	0.102
	mALBI grade 2 vs. 1	0.602 (0.402–0.839	< 0.001
	mALBI grade 3 vs. 1	1.260 (0.997–1.554)	< 0.001
	mALBI grade 3 vs. 2	0.658 (0.389–0.900)	< 0.001

Abbreviations: ALBI, albumin–bilirubin; CI, confidence interval; mALBI, modified albumin–bilirubin; TACE, transarterial chemoembolization

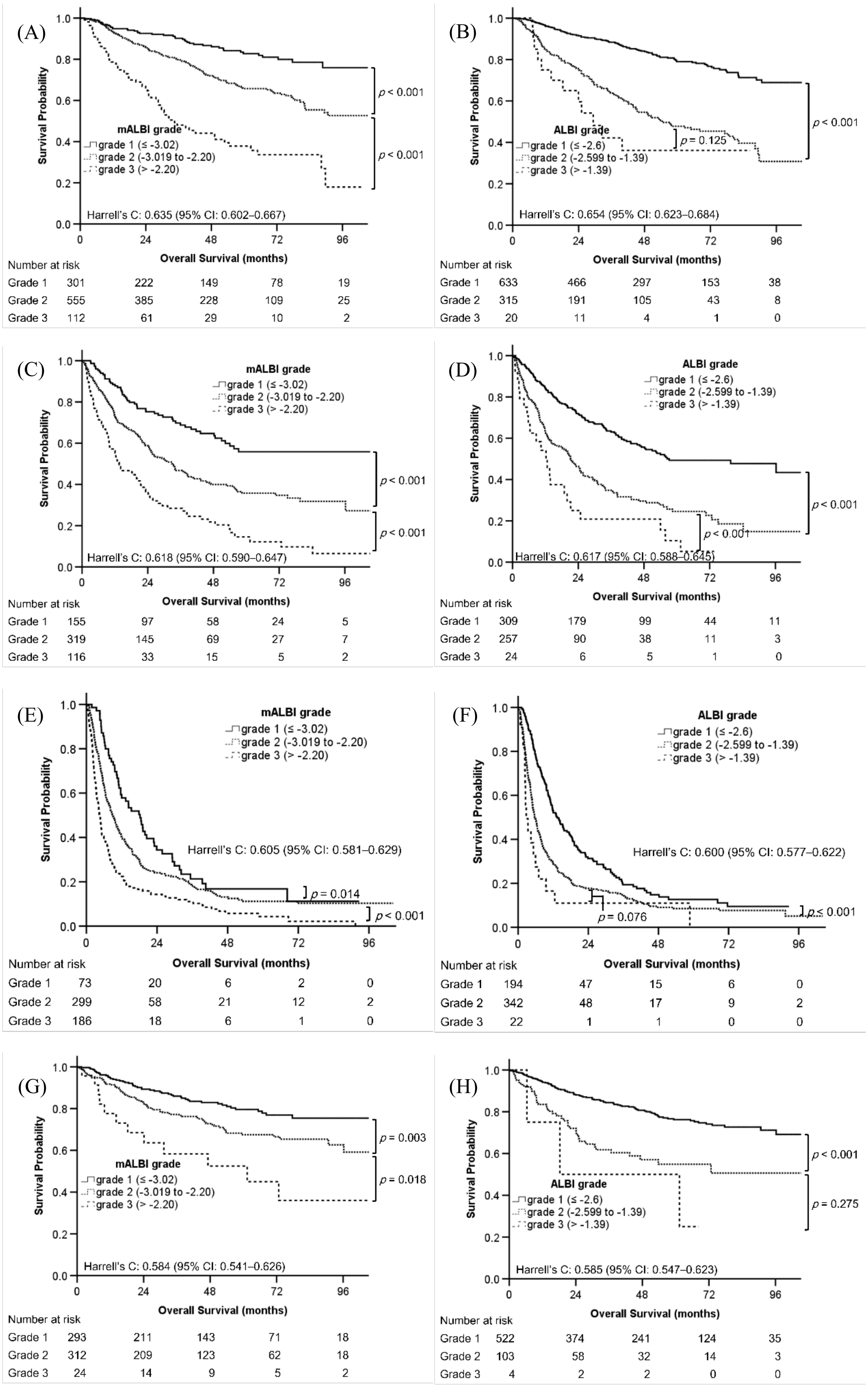

(A)
Survival Probability
mALBI grade
grade 1 (≤ -3.02)
grade 2 (-3.019 to -2.20)
grade 3 (> -2.20)
p < 0.001
p < 0.001
Harrell's C: 0.635 (95% CI: 0.602–0.667)
Overall Survival (months)
Number at risk
Grade 1 301 222 149 78 19
Grade 2 555 385 228 109 25
Grade 3 112 61 29 10 2
(B)
Survival Probability
ALBI grade
grade 1 (≤ -2.6)
grade 2 (-2.599 to -1.39)
grade 3 (> -1.39)
p = 0.125
p < 0.001
Harrell's C: 0.654 (95% CI: 0.623–0.684)
Overall Survival (months)
Number at risk
Grade 1 633 466 297 153 38
Grade 2 315 191 105 43 8
Grade 3 20 11 4 1 0
(C)
Survival Probability
mALBI grade
grade 1 (≤ -3.02)
grade 2 (-3.019 to -2.20)
grade 3 (> -2.20)
p < 0.001
p < 0.001
Harrell's C: 0.618 (95% CI: 0.590–0.647)
Overall Survival (months)
Number at risk
Grade 1 155 97 58 24 5
Grade 2 319 145 69 27 7
Grade 3 116 33 15 5 2
(D)
Survival Probability
ALBI grade
grade 1 (≤ -2.6)
grade 2 (-2.599 to -1.39)
grade 3 (> -1.39)
p < 0.001
p < 0.001
Harrell's C: 0.617 (95% CI: 0.588–0.645)
Overall Survival (months)
Number at risk
Grade 1 309 179 99 44 11
Grade 2 257 90 38 11 3
Grade 3 24 6 5 1 0
(E)
Survival Probability
mALBI grade
grade 1 (≤ -3.02)
grade 2 (-3.019 to -2.20)
grade 3 (> -2.20)
Harrell's C: 0.605 (95% CI: 0.581–0.629)
p = 0.014
p < 0.001
Overall Survival (months)
Number at risk
Grade 1 73 20 6 2 0
Grade 2 299 58 21 12 2
Grade 3 186 18 6 1 0
(F)
Survival Probability
ALBI grade
grade 1 (≤ -2.6)
grade 2 (-2.599 to -1.39)
grade 3 (> -1.39)
Harrell's C: 0.600 (95% CI: 0.577–0.622)
p = 0.076
p ≤ 0.001
Overall Survival (months)
Number at risk
Grade 1 194 47 15 6 0
Grade 2 342 48 17 9 2
Grade 3 22 1 1 0 0
(G)
Survival Probability
mALBI grade
grade 1 (≤ -3.02)
grade 2 (-3.019 to -2.20)
grade 3 (> -2.20)
p = 0.003
p = 0.018
Harrell's C: 0.584 (95% CI: 0.541–0.626)
Overall Survival (months)
Number at risk
Grade 1 293 211 143 71 18
Grade 2 312 209 123 62 18
Grade 3 24 14 9 5 2
(H)
Survival Probability
ALBI grade
grade 1 (≤ -2.6)
grade 2 (-2.599 to -1.39)
grade 3 (> -1.39)
p < 0.001
p = 0.275
Harrell's C: 0.585 (95% CI: 0.547–0.623)
Overall Survival (months)
Number at risk
Grade 1 522 374 241 124 35
Grade 2 103 58 32 14 3
Grade 3 4 2 2 0 0

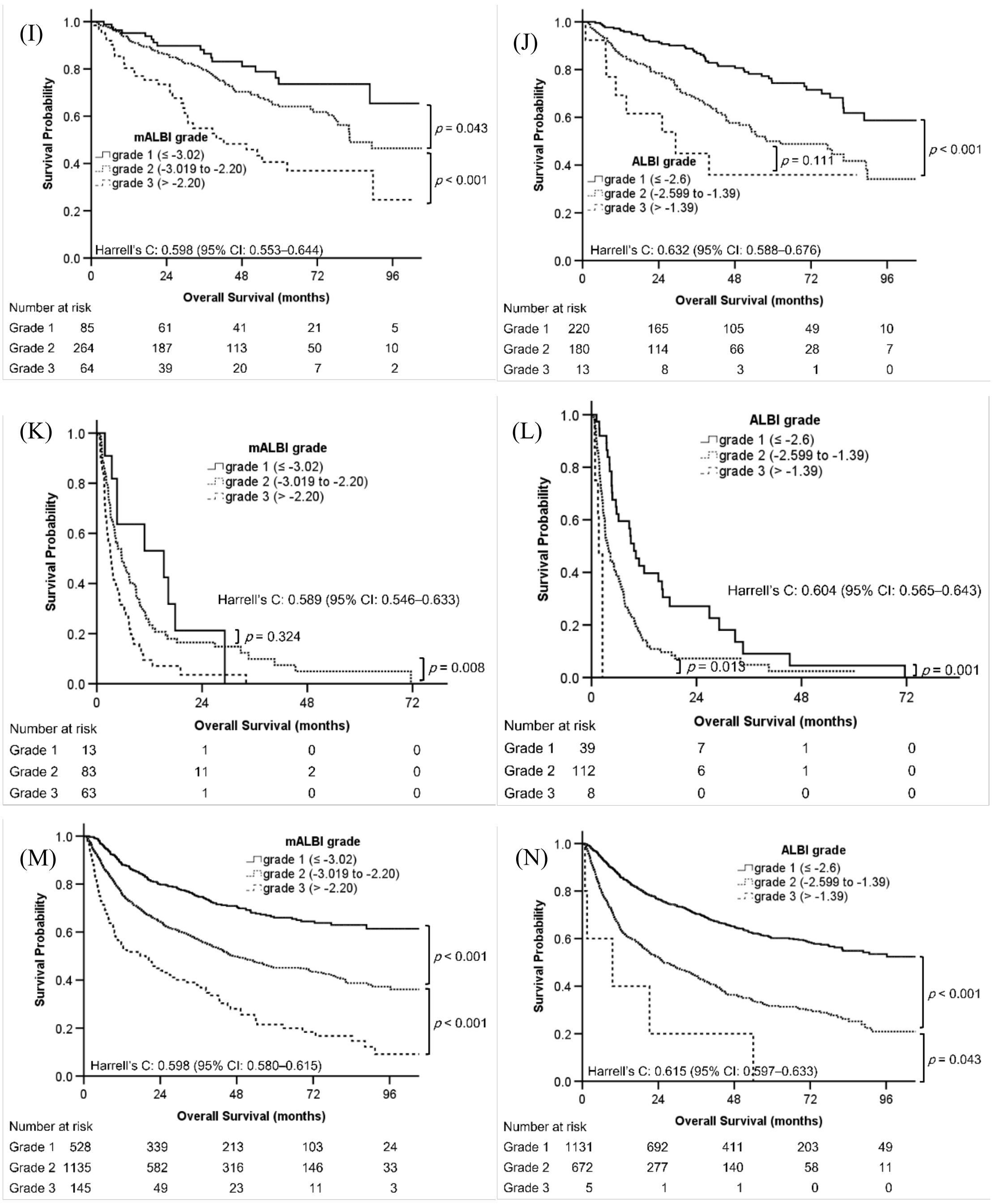

Figure S1. Kaplan–Meier analyses of overall survival. (A) mALBI grade in patients with BCLC A. (B) Original ALBI grade of patients BCLC A. (C) mALBI grade in patients with BCLC B. (D) Original ALBI grade of patients BCLC B. (E) mALBI grade in patients with BCLC C. (F) Original ALBI grade of patients BCLC C. (G) mALBI grade in patients receiving surgical resection. (H) Original ALBI grade of

patients receiving surgical resection. (I) mALBI grade of patients receiving RFA. (J) Original ALBI grade of patients receiving RFA. (K) mALBI grade of patients receiving systemic therapy. (L) Original ALBI grade of patients receiving systemic therapy. (M) mALBI grade of patients with Child–Pugh class A. (N) Original ALBI grade of patients with Child–Pugh class A. ALBI, albumin–bilirubin; CI, confidence interval; mALBI, modified ALBI; RFA, radiofrequency ablation.
